# The physical activity and social support scale: a translation and psychometric validation study in a Chinese college student sample

**DOI:** 10.3389/fpsyg.2024.1252561

**Published:** 2024-04-05

**Authors:** Yunxia Cao, Junfeng Yuan, Lin Luo

**Affiliations:** ^1^School of Physical Education, Guizhou Normal University, Guiyang, China; ^2^Guizhou Vocational College of Sports, Guiyang, China

**Keywords:** physical activity social support scale (PASSS), physical activity, social support, college students, measurement invariance

## Abstract

**Objective:**

This study aims to evaluate the reliability and validity of the Chinese version of the Physical Activity Social Support Scale (PASSS-C) and its measurement invariance across different gender groups in a Chinese college student sample.

**Methods:**

A total of 1,689 Chinese college students participated in the study. We assessed the internal consistency of PASSS-C using Cronbach’s alpha and McDonald’s omega. A Confirmatory Factor Analysis (CFA) was conducted to test its five-factor model. Multi-group CFA was used to examine measurement equivalence between male and female groups. Convergent and criterion-related validity were assessed using Pearson correlation coefficients.

**Results:**

The overall internal consistency of PASSS-C was good with a Cronbach’s alpha of 0.952, and the subscales showed acceptable consistency. The CFA results supported the five-factor structure of PASSS-C in the college student sample, with values of CFI = 0.932, TLI = 0.917, RMSEA = 0.048, 90% CI [0.043 0.053], SRMR = 0.047. Scalar invariance was also supported across different gender groups, with ΔCFI = −0.003, ΔTLI = 0, ΔRMSEA = 0. PASSS-C demonstrated good convergent and criterion-related validity.

**Conclusion:**

PASSS-C exhibits satisfactory psychometric properties and is a valid and reliable tool for assessing the perceived level of social support for physical activity among college students.

## Introduction

Physical activity is universally acknowledged for its extensive health benefits. It plays an indispensable role in preventing chronic diseases and enhancing overall well-being. The World Health Organization (WHO) has identified physical inactivity as a significant global public health issue, linking it to an increased risk of non-communicable diseases and premature mortality (Hallal et al., 2012; World Health Organization, 2020). The prevalence of inactivity varies greatly across countries, with some adult subpopulations experiencing inactivity rates up to 80% (Kaczynski et al., 2011; Loprinzi et al., 2018). This concerning trend highlights the urgent necessity for interventions that encourage physical activity, particularly in groups prone to high levels of inactivity, such as college students.

The psychological dimensions of physical activity, especially among college students, are of considerable interest. This demographic is at a pivotal life stage, where habit formation can profoundly impact long-term health and well-being. Research indicates that college students encounter unique challenges that may hinder physical activity, including academic stress, time constraints, and lifestyle shifts (Maher et al., 2013; Lee et al., 2021). Thus, identifying factors that promote or inhibit physical activity in this population is crucial.

Social support, as defined by Cohen et al., comprises various forms of assistance provided by others and is acknowledged as a vital element in promoting health behaviors, including physical activity (Cohen and Wills, 1985). Studies have consistently demonstrated that higher levels of social support correlate with increased physical activity and greater adherence to exercise regimens (Penedo and Dahn, 2005; Sallis et al., 2015). This support manifests in diverse forms, such as emotional encouragement, companionship, informational support, and instrumental aid (Cohen and Wills, 1985; Cutrona and Russell, 1990).

However, accurately measuring social support in the context of physical activity presents challenges, especially in culturally diverse groups like Chinese college students. There is a clear need for a valid and reliable tool that effectively captures the complex nature of social support in these settings. The Physical Activity Social Support Scale (PASSS), devised by Golaszewski and Bartholomew (2019), addresses this need comprehensively. Based on Cohen’s theory of functional social support, this scale includes all five forms of support and has demonstrated promising results in initial validations (Golaszewski and Bartholomew, 2019).

The relevance of social support in influencing physical activity behaviors is particularly pronounced among Chinese college students. In China, transitioning to college life often entails significant lifestyle alterations that may impact physical activity levels, including new academic pressures, changed living situations, and varied social environments (Chen et al., 2020; Luo et al., 2021). The support from family, friends, and peers is vital in assisting students to navigate these changes and either maintain or elevate their physical activity levels (Mendonça et al., 2014; Koh et al., 2022).

Social support is a complex concept, encompassing dimensions such as emotional, instrumental, informational, and appraisal support (Cohen and Wills, 1985; Cutrona and Russell, 1990). Emotional support involves expressions of empathy, love, trust, and caring. Instrumental support refers to tangible assistance and services provided to those in need. Informational support encompasses the offering of advice, suggestions, and information. Appraisal support includes feedback and affirmation useful for self-evaluation (Procidano and Heller, 1983; Cutrona and Russell, 1990). Each type plays a unique role in influencing physical activity behaviors.

Despite the acknowledged importance of social support in enhancing physical activity, there is a gap in the availability of culturally sensitive measurement tools in China. Existing scales, like the Social Influence Scale and the Social Provisions Scale adapted for physical activity, are predominantly used in Western contexts (Chogahara, 1999; Perera, 2016). These may not fully capture the cultural specifics of the Chinese context, where family dynamics, social norms, and values related to physical activity differ (Ball et al., 2010; Gu et al., 2019).

Hence, adapting the Physical Activity Social Support Scale (PASSS) to the Chinese cultural milieu is essential. The PASSS, incorporating all forms of Cohen’s functional social support, offers a comprehensive framework for assessing the multidimensional nature of social support in physical activities (Golaszewski and Bartholomew, 2019). Adapting it for Chinese college students not only fills a significant gap in existing literature but also provides a valuable resource for researchers and practitioners in this domain.

This study aims to translate and evaluate the psychometric properties of the PASSS within a sample of Chinese college students. This entails assessing the scale’s factor structure, reliability, and validity, including structural and concurrent validity. The study also intends to explore the correlations between social support (measured through the PASSS) and key behavioral outcomes, such as physical exercise behaviors, which are related to both social support and physical activity (Mendonça et al., 2014; Laird et al., 2016). By examining these relationships, this study seeks to gain deeper insights into how social support influences physical activity among Chinese college students. This insight is crucial for devising effective interventions to promote physical activity in this population.

## Methods

### Translation procedure

To ensure the adaptation of the Physical Activity Social Support Scale (PASSS) to the Chinese cultural context, this study utilized Brislin’s back-translation technique (Brislin, 1970). Initially, permission to use the PASSS was obtained from the original authors. The forward translation of the PASSS was undertaken by two bilingual psychology graduate students and an expert in sports psychology. Subsequently, two proficient English-speaking graduate students conducted the initial Chinese version’s back-translation. After comparing the back-translated version with the original PASSS, the initial Chinese version of PASSS-C underwent several revisions by three psychologists and a sports scientist, resulting in the second version. The comprehensibility and acceptability of the second version of PASSS-C were assessed by three students, culminating in the final version. PASSS-C consists of 20 items across five dimensions: emotional support, appraisal support, informational support, companionship support, and instrumental support. The English and Chinese versions of PASSS, along with the scoring rules, are included in Appendix.

### Procedure and participants

This study was approved by the Academic Ethics Committee of Guizhou Normal University. Participants were recruited from several universities in Southwest China. The researchers, based on prior collaborations, recruited survey volunteers from these universities. These volunteers received uniform training and explanations about the study. Additionally, participants were informed that their responses would remain anonymous and that they could withdraw from the study at any time. The inclusion criteria for participants were: Chinese nationality university students without developmental disabilities or physical impairments that limit physical activity. To accurately reflect the prevalence of physical inactivity among university students in China, this study utilized the sample size estimation formula recommended by the World Health Organization (WHO) (Lwanga and Lemeshow, 1991). The formula for calculating the sample size is as follows:


$$n=\frac{Z^{2}P\left(1-P\right)}{d^{2}}\times \mathrm{deff}$$

Here, *n* represents the sample size; *Z* is the *Z*-statistic for the confidence level of 1 − *α*/2; *P* denotes the expected rate; *d* is the precision level (i.e., the allowable absolute error level); and deff stands for the design effect factor. Drawing from the National College Student Physical Fitness and Health Survey results of 2022, this study sets the expected rate of physical inactivity among Chinese university students at *p* = 30% (Ministry of Education, P. R. China, Department of Physical Education, Health and Art Education, 2022). Following epidemiological sampling guidelines, we set a Type I error *α* = 0.05, *Z*1 − α/2 = 1.96, an allowable absolute error level *d* = 5%, and a design effect deff = 2.0. The minimum required sample size is then 645 cases. Additionally, to account for the exclusion of invalid data or data that does not meet the inclusion criteria, a dropout rate of 30% is set. This dictates a minimum required sample size of 921 cases. The questionnaire was ultimately distributed to 1863 university students, with 1,698 completing the full online survey. Demographic characteristics of the participants are shown in Table 1.

**Table 1:** Descriptive information for the present sample (*n* = 1,698).

Variables	Number (percentage)
Gender	
Male	850 (50.08%)
Female	548 (49.92%)
Age(y)	
16	3 (0.16%)
17	16 (0.97%)
18	371 (21.84%)
19	542 (31.9%)
20	493 (29.05%)
21	206 (12.16%)
22	53 (3.12%)
23	6 (0.38%)
24	5 (0.32%)
25	2 (0.10%)
Ethnicity	
Han ethnicity	951 (55.98%)
Ethnic minorities	747 (44.02%)
Household registration	
Rural area	1,295 (76.27%)
Urban area	403 (23.73%)
Grade	
Freshman	830 (48.9%)
Sophomore	855 (50.35%)
Junior	8 (0.48%)
Senior	5 (0.27%)
Major	
Engineering	520 (30.65%)
Arts	293 (17.28%)
Science	487 (28.66%)
Humanities	397 (23.4%)

### Measures

#### Social support for physical activity

The Chinese version of the Physical Activity Social Support Scale (PASSS-C) was employed to assess participants’ perceived social support concerning physical activities. This scale comprises five dimensions: emotional support (4 items), informational support (4 items), instrumental support (4 items), validation support (4 items), and companionship support (4 items), totaling 20 items. The overall PASSS-C score is the sum of all 20 item scores, with scoring options ranging from 1 to 7. The option “does not apply” is scored as 0, and the scores corresponding to the PASSS-C are calculated accordingly. The Cronbach’s alpha coefficient for PASSS-C is 0.942.

#### Social support

The Chinese version of the Social Support Rating Scale (SSRS) (Sun et al., 2020) was used to assess participants’ perceived levels of social support. The scale consists of three dimensions: objective support (3 items), subjective support (4 items), and support utilization (3 items), totaling 10 items. The SSRS total score is the sum of scores for all 10 items, with items 1–4 and 8–10 being single-choice questions, scored on a range from 1 to 4 based on the response options. Item five is a multiple-choice question with response options A, B, C, and D, scored on a range from 1 to 4 depending on the level of support provided. For items six and seven, selecting “no sources” results in a score of 0, while selecting one or more sources will result in corresponding calculations of scores. The Cronbach’s α coefficient for the SSRS is 0.812.

#### Physical activity

The Chinese version of the Physical Activity Rating Scale-3 (PARS-3) was used to assess the levels of self-initiated physical exercise among university students. It includes three items, each scored from 1 to 5, with the formula for calculating the exercise level being: Exercise Level = Intensity * Time * Frequency (Ren et al., 2021). The total score ranges from 0 to 100, with ≤19 indicating a low level of exercise, 20–42 a moderate level, and ≥ 43 a high level of exercise.

### Data analysis

In this study, Stata software (version 26.0) was employed for the descriptive statistical analysis of all scales. To assess the internal reliability of the PASSS-C scale, we computed Cronbach’s alpha (α) and McDonald’s omega (ω) coefficients. According to Cronbach’s α coefficient, consistency levels were categorized as follows: <0.60 indicating low consistency, 0.60–0.69 indicating marginal consistency, 0.70–0.79 indicating acceptable consistency, 0.80–0.89 indicating good consistency, and ≥ 0.90 indicating excellent consistency, as previous research has suggested a minimum acceptable value of 0.60 for α and ω (Tavakol and Dennick, 2011).

To validate the original five-factor structure model of the PASSS-C scale within our study sample, we conducted a confirmatory factor analysis (CFA) using Mplus software (version 8.3) (Muthén and Muthén, 2009). Considering that skewness and kurtosis values for the data slightly exceeded the standard range of −1 to +1 (as detailed in Table 2), we employed robust maximum likelihood estimation (MLM), which is more suitable for non-normally distributed data (Muthén and Muthén, 2009). Model fit was assessed by comparing fit indices, including the Comparative Fit Index (CFI), Tucker-Lewis Index (TLI), and Root Mean Square Error of Approximation (RMSEA). Generally, model fit was considered good when CFI and TLI values exceeded 0.90, and RMSEA values were less than 0.08, with SRMR values also below 0.08 (Hu and Bentler, 1999).

**Table 2:** Means, SDs, skewness, kurtosis, Cronbach’s α and McDonald’s ω and factor loadings of the PASSS-C (*n* = 1,689).

Coding	Item	M	SD	Skewness	Kurtosis	Loading	Internal consistency
	Emotional support						α = 0.925 ω = 0.947
Q1	I have someone who can provide reassurance in the activity/activities	3.726	1.492	0.401	−0.566	0.827	
Q2	There is someone that provides me with positive feedback in the activity/activities.	3.786	1.47	0.346	−0.585	0.818	
Q3	There is someone who understands my problems/worries about the activity/activities.	3.734	1.465	0.394	−0.546	0.824	
Q4	I have someone with whom I can relate to in the activity/activities.	3.686	1.449	0.427	−0.467	0.771	
	Validation support						α = 0.813 ω = 0.877
Q5	I set expectations based on the performance of others in the activity/activities.	3.637	1.355	0.39	−0.384	0.429	
Q6	I want to know competition results (i.e., race results), times, duration, weights, or actions of others in the activity/activities.	3.566	1.424	0.462	−0.338	0.696	
Q7	I compare myself to others in the activity/activities.	3.372	1.333	0.58	−0.076	0.835	
Q8	I use social media to find other people’s performance in the activity/activities to compare to my own.	3.187	1.365	0.646	0.033	0.780	
	Informational support						α = 0.877 ω = 0.916
Q9	I read articles about the activity/activities.	3.499	1.412	0.543	−0.263	0.689	
Q10	I seek out information from others to get better at the activity/activities.	3.64	1.378	0.444	−0.358	0.686	
Q11	I talk to people for assistance or to improve technique in the activity/activities.	3.818	1.412	0.323	−0.515	0.654	
Q12	I attend clinics, classes, and workshops to learn about the activity/activities.	3.472	1.39	0.497	−0.25	0.614	
	Companionship support						α = 0.860 ω = 0.905
Q13	I am part of a core group of people who do the activity/activities.	3.335	1.35	0.469	−0.186	0.518	
Q14	When not engaging in the activity/activities, I still spend time with people that I met while in the activity/activities.	3.568	1.372	0.476	−0.301	0.757	
Q15	I feel a sense of belonging to a group that also does the activity/activities I do.	3.745	1.392	0.369	−0.456	0.602	
Q16	I can find someone to do the activity/activities with, even outside of my friends.	3.56	1.402	0.446	−0.367	0.582	
	Instrumental support						α = 0.875 ω = 0.914
Q17	I can get help traveling if needed to perform the activity/activities.	3.367	1.384	0.507	−0.241	0.666	
Q18	I have someone that could loan or give me something to help carry out the activity/activities I do.	3.491	1.419	0.516	−0.23	0.723	
Q19	I have someone who would watch my child(ren) or pets if needed for me to engage in the activity/activities.	3.336	1.523	0.445	−0.366	0.830	
Q20	I can find someone to help on a short notice so that I can engage in the activity/activities.	3.456	1.425	0.424	−0.301	0.730	
	Total						α = 0.952 ω = 0.914

Additionally, the scale’s structural validity was assessed in terms of convergent and discriminant validity. Average Variance Extracted (AVE) values and Composite Reliability (CR) values were computed to evaluate convergent validity. AVE values exceeding 0.50, although those above 0.40 are also considered acceptable, and CR values greater than 0.70 indicate suitable convergent validity for the scale. For assessing discriminant validity, the square root of the AVE values was calculated and compared with the correlation coefficients among factors. It is necessary for the square root of AVE values to exceed the correlation coefficients between the corresponding factors (Yang et al., 2023).

To ensure that the observed differences in PASSS-C scores reflected true individual variations rather than measurement error, we examined measurement invariance between different gender groups using a CFA-based technique (Meredith, 1993). The gender groups were categorized into female and male groups to investigate potential differences, consistent with previous findings regarding gender disparities in physical activity research. Following established procedures (Millsap, 2012), we first evaluated configural invariance to ensure equivalence of factor structure across different groups, with all observed variable parameters freely estimated. Subsequently, based on these results, we further assessed metric invariance, testing the consistency of factor loadings between different groups. In metric invariance analysis, factor loadings were constrained to be equal to ensure item consistency across groups. Metric invariance was considered established when the fit of the metric model did not significantly differ from that of the configural model. In the third step, we evaluated scalar invariance by constraining item thresholds to be equal across groups. Scalar invariance was considered established when the fit of the scalar model did not significantly differ from that of the metric model. Finally, we examined strict invariance to ascertain the equivalence of error variances across groups. Strict invariance was established when the fit of the strict model did not significantly differ from that of the scalar model. According to previous recommendations (Millsap, 2012), ΔCFI <0.010, ΔTLI <0.010, and ΔRMSEA <0.015 indicated measurement invariance. Independent sample *t*-tests were conducted to examine gender differences in PASSS-C scores only after establishing measurement invariance. Independent sample *t*-tests were employed to compare mean differences in both total PASSS-C scores and scores on individual subscales between different groups.

Furthermore, Pearson correlation coefficients were calculated to assess the convergent and discriminant validity of the PASSS-C scale. According to established guidelines, correlation coefficients between 0.10 and 0.30 were indicative of weak correlations, coefficients between 0.30 and 0.50 indicated moderate correlations, and coefficients greater than 0.50 signified strong correlations.

## Results

### Reliability

In this assessment, the overall PASSS-C scale demonstrated strong internal consistency, with a Cronbach’s alpha (α) value of 0.952 and a McDonald’s omega (ω) value of 0.914, indicating excellent internal reliability. Among the various subscales, the Emotional Support subscale exhibited an α value of 0.925 and an ω value of 0.947. The Validation Support subscale had an α value of 0.813 and an ω value of 0.877. The Informational Support subscale yielded an α value of 0.877 and an ω value of 0.916. The Companionship Support subscale achieved an α value of 0.860 and an ω value of 0.905, while the Instrumental Support subscale obtained an α value of 0.875 and an ω value of 0.914. These results collectively demonstrate that both the overall scale and its individual subscales exhibit satisfactory reliability and validity in measuring relevant psychological dimensions (see Table 2).

### Structure validity

**Figure 1:**
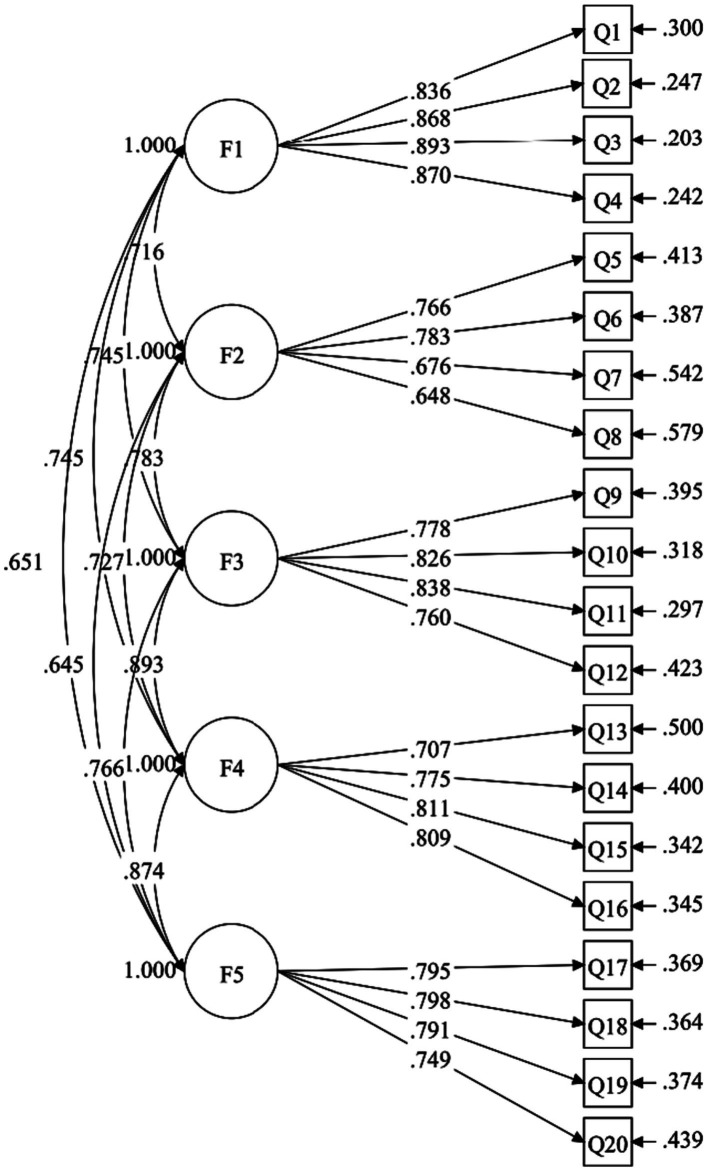
Confirmatory factor analysis model of the five-factor PASSS-C. Standardized path coefficients were presented in this model. F1, Emotion support; F2, Validation support; F3, Information support; F4, Companionship support; F5, Instrumental support.

This study directly examined the five-factor model of PASSS-C, which is consistent with the original research and the structure of PASSS. The results indicated that the five-factor model demonstrated a good fit: CFI = 0.932, TLI = 0.917, RMSEA = 0.048, 90% CI [0.043 0.053], SRMR = 0.047. In the Confirmatory Factor Analysis (CFA) model (see Figure 1), the factor loadings for Emotional Support ranged from 0.771 to 0.827, Validation Support ranged from 0.429 to 0.835, Informational Support ranged from 0.614 to 0.689, Companionship Support ranged from 0.518 to 0.757, and Instrumental Support ranged from 0.666 to 0.830 (see Table 2). These results collectively demonstrate that PASSS-C exhibits strong structural validity in measuring relevant psychological dimensions.

### Measurement invariance

Multi-Group Confirmatory Factor Analysis (MCFA) was employed to examine the measurement invariance between males and females in the context of PASSS-C (Perceived Availability of Social Support—Chinese version). The fit indices for the configural invariance model (CFI = 0.928, TLI = 0.912, RMSEA = 0.050) indicated the successful establishment of configural equivalence. Consequently, this model was adopted as the baseline for subsequent analyses. Satisfactory fit indices (ΔCFI = 0, ΔTLI = +0.005, ΔRMSEA = −0.002) supported the measurement invariance model. The fit indices for the scalar invariance model also demonstrated satisfactory results (ΔCFI = −0.003, ΔTLI = 0, ΔRMSEA = 0). However, the fit indices for the strict invariance model were as follows: ΔCFI = −0.014, ΔTLI = −0.009, and ΔRMSEA = +0.003 (refer to Table 3), indicating a lack of support for strict measurement invariance. Therefore, these results provide evidence for the measurement invariance of PASSS-C, at least at the scalar level, between male and female groups.

**Table 3:** Model comparisons for measurement invariance testing across male and female groups.

Model	CFI	TLI	RMSEA	ΔCFI	ΔTLI	ΔRMSEA	Decision
Model 1	0.928	0.912	0.050				
Model 2	0.928	0.917	0.048	0	0.005	−0.002	Accept
Model 3	0.925	0.917	0.048	−0.003	0	0	Accept
Model 4	0.911	0.908	0.051	−0.014	−0.009	0.003	Reject

Model 1, configural invariance; Model 2, metric invariance; Model 3, scalar invariance; Model 4, residual invariance; TLI, Tucker-Lewis index; CFI, comparative fit index; RMSEA, root-mean-square error of approximation; ΔCFI, ΔTLI, and ΔRMSEA values represent comparisons of in-row model with model immediately above the row.

Subsequently, independent sample *t*-tests revealed that the total PASSS-C scores for the female group were significantly higher than those for the male group. Specifically, the female group scored significantly higher than the male group on all four subscales of PASSS-C, namely, Instrumental support, Informational support, Validation support, and Emotional support (see Table 4).

**Table 4:** Means, standard deviations (mean ± SD) and between sample differences for PASSS-C (Male: *N* = 933; Female: *N* = 930).

	Male	Female	*t*-value	*p*-value
Instrumental support	13.31 ± 5.05	13.79 ± 4.74	−2.066	0.039
Companionship support	14.06 ± 4.91	14.22 ± 4.32	−0.769	0.442
Informational support	14.13 ± 5.02	14.63 ± 4.52	−2.224	0.026
Validation support	13.53 ± 4.53	13.85 ± 4.24	−1.587	0.113
Emotional support	14.19 ± 5.42	15.61 ± 5.13	−5.764	<0.001
PASSS-C	69.14 ± 21.77	71.91 ± 19.14	−2.895	0.004

### Convergent and divergent validity

In this analysis of convergent validity, it was observed that the Average Variance Extracted (AVE) values for all five factors exceeded 0.5, while their Composite Reliability (CR) values were above 0.7. These data indicate (as shown in Table 5) that the data in the analysis possess excellent aggregation validity, i.e., good convergent validity. Further analysis was conducted on discriminant validity: For Emotional support, the square root of its AVE value was 0.870, higher than the maximum absolute value of the correlation coefficients between any two factors, which was 0.671, demonstrating significant discriminant validity. Similarly, for Validation support, the square root of the AVE was 0.728, exceeding the highest absolute value of correlation coefficients between factors, 0.653, indicating good discriminant validity. For Informational support, the square root of the AVE was 0.802, also greater than the highest absolute value of the correlation coefficients between factors, 0.783, further confirming its good discriminant validity. For Companionship support, the square root of the AVE was 0.781, slightly above the highest absolute value of correlation coefficients between factors, 0.780, showing similarly good discriminant validity. Finally, for Instrumental support, the square root of the AVE was 0.801, surpassing the highest absolute value of correlation coefficients between factors, 0.764, reaffirming its excellent discriminant validity. In summary, the data in this analysis demonstrated good performance in both convergent and discriminant validity.

**Table 5:** Convergent validity and discriminant validity of the scale.

	1	2	3	4	5	AVE	Sqrt (AVE)	CR
1.Emotional support	1.000					0.757	0.870	0.926
2.Validation support	0.575	1.000				0.530	0.728	0.818
3.Informational support	0.671	0.653	1.000			0.644	0.802	0.878
4.Companionship support	0.657	0.525	0.780	1.000		0.611	0.781	0.862
5.Instrumental support	0.584	0.529	0.676	0.764	1.000	0.642	0.801	0.878

### Criterion-related validity

The data reveals (Table 6) a significant positive correlation between the various subscales of PASSS-C (Instrumental support, Companionship, Informational support, Validation support, and Emotional support) and the total SSRS (Social Support Rating Scale) score. Specifically, the total PASSS-C score exhibits a notably high correlation with SSRS scores (*r* = 0.949), indicating a strong relationship among these indicators of social support. The significant correlations between the PASSS-C subscales and SSRS demonstrate robust convergent validity for PASSS-C. This can be substantiated by its consistency with theoretical expectations, suggesting that measures assessing similar constructs (in this case, various aspects of social support) should exhibit a high degree of correlation. Table 7 correlation coefficients show that there is a significant correlation between various subscales of PASSS-C and physical activity.

**Table 6:** Correlations between the PASSS-C and the SSRS.

	1	2	3	4	5	6	7	8	9
1.Instrumental support	1								
2.Companionship support	0.758**	1							
3.Informational support	0.670**	0.777**	1						
4.Validation support	0.526**	0.587**	0.655**	1					
5.Emotional support	0.583**	0.656**	0.669**	0.579**	1				
6.PASSS-C	0.837**	0.891**	0.889**	0.783**	0.833**	1			
7.Objective support	0.139**	0.174**	0.181**	0.126**	0.225**	0.199**	1		
8.Subjective support	0.319**	0.337**	0.320**	0.212**	0.401**	0.387**	0.383**	1	
9.Support utilization	0.299**	0.353**	0.321**	0.230**	0.374**	0.373**	0.216**	0.405**	1
10.SSRS	0.358**	0.394**	0.372**	0.252**	0.456**	0.442**	0.491**	0.949**	0.652**

*p < 0.05; **p < 0.01.

**Table 7:** Correlations between the PASSS-C and physical activity.

	1	2	3	4	5	6
1.Instrumental support	1					
2.Companionship support	0.574**	1				
3.Informational support	0.477**	0.568**	1			
4.Validation support	0.366**	0.399**	0.474**	1		
5.Emotional support	0.439**	0.492**	0.503**	0.384**	1	
6.PASSS-C	0.650**	0.705**	0.701**	0.571**	0.664**	1
7.Physical activity	0.073*	0.112**	0.104**	0.041	0.104**	0.116**

*p < 0.05; **p < 0.01.

## Discussion

The PASSS-C, a newly developed self-report scale, is specifically designed to assess university students’ perceptions of social support for physical activity. This study emphasized evaluating the psychometric properties of PASSS-C, particularly focusing on measurement invariance across genders in a university student sample. Our results demonstrate that PASSS-C exhibits strong reliability and validity and maintains measurement invariance across genders in university student samples. This supports its suitability as a tool for assessing university students’ social support levels for physical activity. The original PASSS-C scale exhibited a high Cronbach’s alpha coefficient of 0.90, indicating substantial reliability. The subscales, including companionship, emotional, informational, instrumental, and validation support, had Cronbach’s alpha coefficients of 0.81, 0.89, 0.81, 0.71, and 0.79, respectively. In this study, PASSS-C’s internal consistency was deemed acceptable, with an overall Cronbach’s alpha of 0.952, showing satisfactory stability. Although the original study did not detail the reliability of the scale and its subscales, we further examined their reliability. Three subscales displayed alpha and omega values above 0.800. Our results indicate that all five dimensions of PASSS-C consist of reasonably homogeneous items, confirming PASSS-C as a scale with acceptable reliability.

Regarding structural validity, the original PASSS-C scale’s confirmatory factor analysis (CFA) showed good model fit, specifically RMSEA = 0.04, 90% CI [0.03, 0.05], *p* = 0.93; CFI = 0.97; TLI = 0.96; SRMR = 0.06. Our study’s CFA analysis supported the five-factor structure of PASSS-C in the university student sample (CFI = 0.932, TLI = 0.917, RMSEA = 0.048, 90% CI [0.043, 0.053], SRMR = 0.047). This result aligns with the original study and may indicate the stability of PASSS-C’s five-factor model across different cultural contexts, suitable for use in both Eastern and Western countries. In our study, PASSS-C demonstrated a stable five-factor structure, further proving its structural validity in China.

Before comparing the scores of different gender groups, it was necessary to assess the measurement invariance of the scale. Therefore, we evaluated the measurement invariance of PASSS-C in male and female groups, including configural, metric, scalar, and strict invariance. The assessment of configural invariance showed that the number and pattern of factors are equivalent across gender groups. The metric invariance assessment indicated that the observed items and latent factors of the tool are equal across different gender groups. The scalar invariance assessment revealed that cross-group differences in observed variable means reflect differences in latent variable means across groups. However, the assessment of strict invariance showed that error variances were not equivalent across each group in this study. Most previous empirical studies consider the assessment of strict invariance to be too stringent and unrealistic (Milfont and Fischer, 2010). Overall, our findings support that the constructs measured by PASSS-C have the same meaning across different gender groups and can provide accurate information for intergroup comparisons. Since we confirmed the equivalence of factor loadings and intercepts in the measurement invariance analysis between male and female groups, we concluded that the level of social support for physical activity is higher in the female group than in the male group in this university student sample, consistent with previous research (Maciaszek et al., 2020).

As per the description provided in Table 5, all five factors in the study exhibit Average Variance Extracted (AVE) values greater than 0.5 and Composite Reliability (CR) values above 0.7. This indicates strong convergent validity for the analyzed data. Convergent validity is achieved when the AVE of a construct is greater than 0.5, suggesting that more than half of the variance observed in the items is due to the construct itself. Similarly, CR values above 0.7 indicate that the construct is reliably measured by its indicators.

The discriminant validity, assessed through the comparison of the square root of AVE with the inter-factor correlations, shows varied results across the constructs. For Emotional Support, the square root of the AVE (0.870) exceeds the maximum absolute inter-factor correlation (0.671), indicating significant discriminant validity. This suggests that Emotional Support is a distinct construct, adequately separated from others in the model. For Validation Support, its AVE square root value (0.728) surpasses the maximum inter-factor correlation absolute value (0.653), signifying good discriminant validity.

However, a potential issue arises with Informational Support and Companionship Support, where the AVE square root values (0.802 and 0.781, respectively) are only marginally higher than their maximum inter-factor correlation absolute values (0.783 and 0.780, respectively). Although this minimal difference could raise questions about the distinctiveness of these constructs, it is important to note that they still meet the Fornell-Larcker criterion for discriminant validity. This suggests that, despite the close values, Informational Support and Companionship Support are sufficiently distinct constructs within the model. It’s also crucial to emphasize that these slight overlaps do not undermine the overall convergent validity of the constructs. The AVE values for both Informational Support and Companionship Support are well above the threshold of 0.5, clearly indicating that a significant proportion of the variance in the observed variables is explained by the constructs themselves. This robust demonstration of convergent validity suggests that the constructs are well-defined and effectively captured by their respective indicators. Moreover, the fact that the CR values for these constructs are above 0.7 further reinforces their reliability and the internal consistency of the measures. This high level of composite reliability, coupled with satisfactory levels of AVE, provides strong evidence of the soundness of the constructs in terms of both convergent and discriminant validity. In summary, while the discriminant validity of Informational Support and Companionship Support shows a closer relationship than ideally desired, this proximity does not significantly detract from the overall validity of the constructs. The constructs still demonstrate a strong degree of distinctiveness and contribute meaningfully to the structural equation model. This ensures that the interpretations and conclusions drawn from the model are based on solid and reliable construct definitions, thereby maintaining the integrity and robustness of the research findings.

Overall, PASSS-C is significantly correlated with SSRS and its subscales, proving the validity of measuring adolescent social support for physical activity through PASSS-C. The correlation between PASSS-C and the level of physical activity further indicates that the level of social support for physical activity is related to the physical activity level of university students, consistent with previous research findings (Trost et al., 2002).

However, our study has limitations. First, it did not use a random sampling method but used a convenience sample of university students from Southwest China. Therefore, it is unclear whether the current results are applicable to other regions of China. Future research should replicate these findings in other regions of China. Second, due to resource limitations, we were unable to include clinical participant samples to explore the psychometric properties of PASSS-C in this group. Therefore, it is necessary to perform the same validation in clinical samples, such as testing the measurement invariance of PASSS-C, to ensure consistency of comparisons. Further research including clinical participants, such as studies on other young disease populations, is necessary. Despite these limitations, our study provides important evidence for the psychometric assessment of PASSS-C in a large sample of Chinese university students and explores and verifies for the first time the measurement invariance of PASSS-C between different gender groups, laying the groundwork for intergroup comparisons.

## Conclusion

In conclusion, PASSS-C demonstrates robust psychometric properties among Chinese university students, serving as an effective and reliable questionnaire to assess their perceived level of social support for physical activity. This endeavor enriches the psychometric attributes of PASSS and holds significant implications for empirical research aimed at enhancing physical activity among university students.

## Data availability statement

The original contributions presented in the study are included in the article/supplementary material, further inquiries can be directed to the corresponding author.

## Ethics statement

The studies involving humans were approved by the Research Ethics Committee of Guizhou Normal University. The studies were conducted in accordance with the local legislation and institutional requirements. The participants provided their written informed consent to participate in this study.

## Author contributions

YC and LL contributed to the original manuscript preparation, experimental design, investigation, data analysis, and writing. JY assisted in reviewing the literature, editing, and administering questionnaires. All authors contributed to the article and approved the submitted version.

## Appendix

**Table A1:** PASSS Chinese and English item comparison table.

Item	English	中文
1	I have someone who can provide reassurance in the activity/activities	当我在活动中感到疑惑、害怕或不适时, 有人能给予我肯定、支持与鼓励。
2	There is someone that provides me with positive feedback in the activity/activities.	在活动过程中, 我会收到他人的积极反馈。
3	There is someone who understands my problems/worries about the activity/activities.	他人能够理解我在活动中所面临的问题和担忧。
4	I have someone with whom I can relate to in the activity/activities.	在活动中, 有人能与我产生共鸣, 因为他们有过类似的经历。
5	I set expectations based on the performance of others in the activity/activities.	我会根据他人的表现来调整自己的期望和目标。
6	I want to know competition results (i.e., race results), times, duration, weights, or actions of others in the activity/activities.	我对其他参与者在活动中的成绩、频次、时长、强度或动作很感兴趣。
7	I compare myself to others in the activity/activities.	在活动中, 我常常将自己与他人进行比较。
8	I use social media to find other people’s performance in the activity/activities to compare to my own.	我会在社交媒体或网络上查看他人的活动表现, 并与自己进行比较。
9	I read articles about the activity/activities.	我经常在杂志、报纸、网络或社交媒体上阅读关于活动的信息。
10	I seek out information from others to get better at the activity/activities.	我从他人那里获得信息或建议, 以提高自己的表现。
11	I talk to people for assistance or to improve technique in the activity/activities.	我通过与他人交流来获得帮助或提升技能。
12	I attend clinics, classes, and workshops to learn about the activity/activities.	为了深入了解某项活动, 我参加相关的短期培训、课程或研讨会。
13	I am part of a core group of people who do the activity/activities.	我是团队活动的核心成员。
14	When not engaging in the activity/activities, I still spend time with people that I met while in the activity/activities.	即使不参加活动, 我也会与活动中认识的朋友们相聚。
15	I feel a sense of belonging to a group that also does the activity/activities I do.	在团队活动中, 我感受到了归属感。
16	I can find someone to do the activity/activities with, even outside of my friends.	即使不是我的朋友, 我也能找到一起参加活动的伙伴。
17	I can get help traveling if needed to perform the activity/activities.	当我需要参加活动时, 总有人能送我到达(例如开车送我去活动场地)。
18	I have someone that could loan or give me something to help carry out the activity/activities I do.	有人会在经济(如借钱)或物质(如提供装备)方面支持我参加活动。
19	I have someone who would watch my child(ren) or pets if needed for me to engage in the activity/activities.	如果我需要参加活动, 总有人帮我照顾孩子或宠物。
20	I can find someone to help on a short notice so that I can engage in the activity/activities.	当我在活动中遇到不确定、恐惧或不适时, 总有人给我肯定、支持和鼓励。
